# Differential Utilization Patterns of Total Ankle Arthroplasty vs Arthrodesis: A United States National Ambulatory Database Analysis

**DOI:** 10.1177/24730114231218011

**Published:** 2023-12-21

**Authors:** Michael R. Mercier, Philip P. Ratnasamy, Nicholas S. Yee, Brandon Hall, Christopher Del Baso, Mohammed Athar, Timothy R. Daniels, Mansur M. Halai

**Affiliations:** 1 Division of Orthopaedics, Department of Surgery, University of Toronto, Toronto, ON, Canada; 2 Department of Orthopaedics and Rehabilitation, Yale School of Medicine, New Haven, CT, USA; 3 Victoria Hospital, Division of Orthopaedic Surgery, University of Western Ontario, London, ON, Canada; 4 St. Michael’s Hospital, Division of Orthopaedic Surgery, University of Toronto, Toronto, ON, Canada

**Keywords:** arthroplasty, arthrodesis, ankle osteoarthritis, utilization patterns, health care disparities

## Abstract

**Background:**

End-stage ankle osteoarthritis is a condition that can be treated with ankle arthrodesis (AA) or total ankle arthroplasty (TAA). The goal of this study is to estimate the 2016-2017 United States’ utilization of TAA and AA in specific ambulatory settings and delineate patient and hospital factors associated with the selection of TAA vs AA for treatment of ankle osteoarthritis.

**Methods:**

TAA and AA procedures performed for ankle osteoarthritis were identified in the 2016-2017 Nationwide Ambulatory Surgery Sample (NASS) Database. Notably, the NASS database only examines instances of ambulatory surgery encounters at hospital-owned facilities. As such, instances of TAA and AA performed at privately owned or freestanding ambulatory surgical centers or those performed inpatient are excluded from this analysis. Cases were weighted using nationally representative discharge weights. Univariate analyses and a combined multiple logistic regression model were used to compare demographic, hospital-related, and socioeconomic factors associated with TAA vs AA.

**Results:**

In total, 6577 cases were identified, which represents 9072 cases after weighting. Of these, TAA was performed for 2233 (24.6%). Based on the logistic regression model, several factors were associated with increased utilization of TAA vs AA. With regard to patient factors, older patients were more likely to undergo TAA, as well as females. Conversely, patients with a higher comorbidity burden were less likely to receive TAA over AA.

With regard to socioeconomic factors, urban teaching and urban nonteaching hospitals were significantly more likely to use TAA compared to rural hospitals. Similarly, privately insured patients and those with a median household income of $71 000 or more were also more likely to receive TAA over AA. Private hospitals (“not-for-profit” and “investor-owned”) were significantly more likely to offer TAA over AA.

**Conclusion:**

Using a large nationally representative cohort, the current data revealed that during 2016-2017, 24.6% of operatively treated cases of end-stage ankle osteoarthritis in the ambulatory setting are treated with TAA. Associations between socioeconomic and hospital-level factors with TAA utilization suggest that nonclinical factors may influence surgical treatment choice for ankle osteoarthritis.

**Level of Evidence:**

Level III, retrospective cohort study.

## Introduction

Osteoarthritis is a degenerative joint disease that causes damage to articular cartilage, subarticular bone remodeling, osteophyte generation, and numerous other pathologic changes to the joint.^19^ In particular, osteoarthritis of the ankle is a common problem, affecting roughly 1% of the entire world adult population.^1^ Ankle osteoarthritis can severely affect patients’ quality of life through impaired mobility, pain, and dysfunction in activities of daily living.^34^ Patients with symptomatic, end-stage ankle arthritis in which conservative measures have failed are often referred for surgical treatment with ankle arthrodesis (AA), or more recently, total ankle arthroplasty (TAA).

Historically, AA has been viewed as the most accepted surgical intervention for definitive treatment of end-stage ankle arthritis.^21^ Despite this, recent advances in technology and surgical techniques regarding TAA have led to improved clinical results and thereby increased popularity of the procedure.^21^ Early studies have shown TAA to have higher or lower rates of major postoperative complications than AA; however, TAA recipients tend to have superior functional outcomes with improved pain.^3,18,22,26^ These studies were of relatively high-quality evidence, ranging from sizeable retrospective cohort studies to smaller prospective comparative analysis and controlled trials. Additionally, TAA patients exhibit significantly higher postoperative arc of motion, whereas AA patients show significantly higher arc of motion at the talonavicular joint, predisposing to subsequent injury.^22^

Selecting the optimal treatment modality for patients with end-stage ankle arthritis is a multifaceted process, with surgeons considering tissue vascularity, patient comorbidities, and physical demands.^21^ In addition to these clinical factors, prior studies in orthopaedics have revealed that other patient demographic factors—such as race, income, insurance status, and others—may influence which treatment modalities are ultimately selected.^28,37^ Similar disparities in patient care have been identified in several elective orthopaedic surgeries, including lumbar spinal fusion, carpal tunnel release, and arthroplasty.^4,6,15,30^

A scarcity of literature exists regarding the influence of sociodemographic factors on whether patients receive AA or TAA. Given this, the present study set out to characterize nationwide utilization of AA and TAA and determine patient, socioeconomic, and hospital factors that influenced the choice of one treatment modality over the alternative. We hypothesize that several, previously unexplored factors, such as hospital location, facility academic status, and patient socioeconomic status may independently influence the likelihood of TAA utilization.

## Methods

### Study Population

The current study used data from the Nationwide Ambulatory Surgery Sample (NASS). Since its release in 2020, NASS represents the largest all-payer ambulatory surgery data set in the United States and represents more than 15 million ambulatory surgical encounters after weighting. Created through a federal-state-industry collaboration and sponsored by the Agency for Healthcare Research and Quality, NASS includes ambulatory surgical encounters from 32 partner states and more than 2500 facilities. The database employs a national sampling scheme that is designed to provide nationally representative estimates of ambulatory surgery encounters across US surgical centers.^12^ Discharge-level weights assigned to each observation in the data set were applied to create national estimates for all analyses, per NASS database documentation.^13^ Surgeries that involve 1 or more overnight stays do not qualify as ambulatory surgical encounters and are thereby not included in the NASS data set. Importantly, the NASS database only examines instances of ambulatory surgery encounters at hospital-owned facilities. As such, procedures performed at privately owned or freestanding ambulatory surgical centers are excluded from this analysis.

NASS is a limited data set, which is defined as health care data in which a certain number of direct identifiers have been removed. Under the Health Insurance Portability and Accountability Act (HIPAA), review by an institutional review board (IRB) is not required for use of limited data sets such as NASS.

### Inclusion and Exclusion Criteria

Data from the 2016-2017 NASS data set were queried for cases of TAA and AA using the following *Current Procedural Terminology* (*CPT*) codes: 27700 and 27702 (TAA) and 27870 and 29899 (TA). Cases were cross-queried for a concurrent *International Classification of Diseases, Tenth Revision, Clinical Modification* (*ICD-10-CM*) diagnosis specifying primary, posttraumatic, or secondary osteoarthrtis of the foot and ankle based on the following codes: M19.07, M19.071, M19.072, M19.079, M19.17, M19.171, M19.172, M19.179, M19.27, M19.271, M19.272, and M19.279. Patients without an explicit *ICD-10-CM* code specifying one of the aforementioned types of ankle osteoarthritis were excluded from the study. All 30 *CPT* code and 15 *ICD-10-CM* code fields were included for analysis. The unit of analysis for the present study was patient visits rather than individual patients given the deidentified nature of the data analyzed.

**Figure 1.**
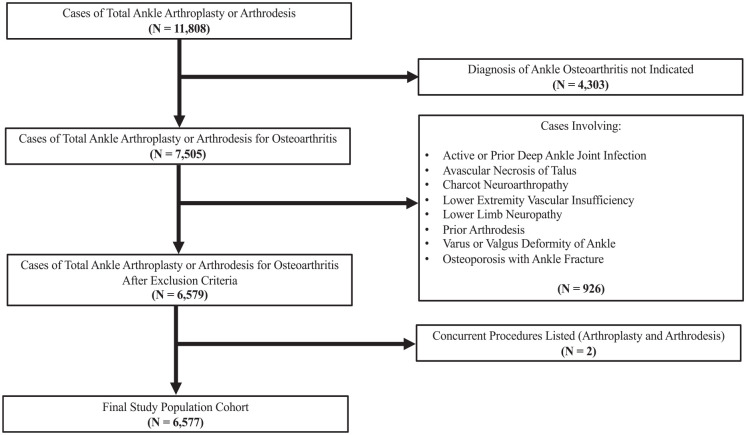
Flowchart depicting selection of the final study population cohort after applying inclusion and exclusion criteria. In total, 6577 cases met inclusion and exclusion criteria, which represented 9072 cases after weighting.

Patients with any of the following documented foot/ankle deformities or comorbidities that may serve as a contraindication to TAA were excluded from the anlaysis: active or prior deep ankle joint infection, avascular necrosis of the talus, Charcot neuroarthropathy, lower extremity vascular insufficiency, lower limb neuropathy, prior arthrodesis, varus or valgus ankle deformity, or significant osteoporosis with a concurrent ankle fracture. A summary of these inclusion and exclusion criteria can be seen in Figure 1.

### Study Variables

Age and sex were extracted from the data set. Overall comorbidity burden of each case was approximated using Elixhauser comorbidity index (ECI). Importantly, NASS does not allow for linkage of succssive claims for the same patient over time. As such, ECI in the present study is only based on visit-level claims rather than on examination of claims history—thus potentially underestimating patients’ overall comorbidity burden. ECI has been used widely in surgical database research as a marker for comorbidity burden and correlates with incidence of postoperative adverse events.^20,29^ A previously validated coding script was used to generate ECI from documented case comorbidites.^32^

Socioeconomic variables analyzed in the data set included payor type (private insurance, Medicaid, and other) and median income quartile based on zip code in which patients reside ($1-$42 999, $43 000-$53 999, $54 000-$70 999, $71 000 or more). Hospital variables analyzed in the data set include hospital bed size (00-99, 100-299, ≥300 beds), hospital type (rural, urban nonteaching, urban teaching) and hospital region (Northeast, Midwest, South, West), and hospital ownership (government, nonfederal; private, not-for-profit; and private, investor-owned). Finally, total charges associated with each encounter were extracted from the data set. Total charges did not include professional fees and were adjusted for inflation.^12^ “Other” payor type, as defined by the NASS database, included worker’s compensation, TriCare, Civilian Health, and Civilian Health and Medical Program of the Uniformed Services (CHAMPUS), CHAMPVA, Title V, and other government programs.^12^

### Statistical Analysis

All statistical analyses were performed using IBM SPSS Statistics, version 28 (IBM, Armonk, NY). To adjust for multiple comparisons made on univariate analysis (n = 6), a Bonferroni correction was applied and statistical significance was set at *P* = .008, and 95% CIs were reported. Significance for goodness-of-fit analysis using the Hosmer-Lemeshow test was set at *P* >.05.

Nationally representative estimates were calculated using weighting factors associated with each discharge encounter. This was accompished using the “weight cases” function in SPSS. Univariate analyses of patient, demographic, socioeconomic, and hospital-level characteristics were conducted using χ^2^ analyses for categorical data, and Student *t* test for continous data. χ^2^ *P* values represent differences in rates of TAA vs AA for each categorical factor of interest. Cases with missing data were omitted from statistical testing calculations, rather than applying an imputation method.

A binary logistic regression model was used to assess the independent effects of each studied covariate on utilization of TAA in the weighted NASS sample. An aggregated model was constructed that included the following factors: patient age (per decade), sex, cumulative ECI, hospital bed size, hospital type, hospital region, payer type, median household income, and hospital ownership type. This model controlled for all factors included in analyzing individual factors’ correlation with TAA utilization. The Hosmer-Lemeshow test was used to determine the goodness of fit for the multivariate logistic regression model. Collinearity was assessed by checking the variance inflation factor for each covariate in the regression model.

## Results

### Study Sample

Between the years 2016 and 2017, there were 6577 unweighted ambulatory surgeries that met our inclusion criteria for isolated surgical treatment of ankle osteoarthritis, which represented 9072 surgeries after weighting. Of these 9072 cases, TAA was performed for 2233 (24.6%), whereas AA was performed for 6839 (75.4%) of them (Table 1).

**Table 1.** Basic Demographic Characteristics of Patients Undergoing Ankle Arthrodesis vs Total Ankle Arthroplasty for Osteoarthritis.

Variables	Total (N = 9072; 100%)	Arthroplasty (TAA) (n = 2233; 24.6%)	Arthrodesis (AA) (n = 6839; 75.4%)	*P* Value
Age, y, mean ± SD	57.12 ± 13.06	56.90 ± 13.6	57.81 ± 11.4	.002
Sex (Female), %	100	45.7	42.7	.014
Elixhauser comorbidity index ≥3, %	17.6	15.1	18.5	<.001

Patients undergoing TAA were older than those undergoing AA (mean 57.81 years [SD 11.4] vs 56.90 years [SD 13.6], *P* = .002), and were more female (45.7% vs 42.7%, *P* = .014). Patients who underwent TAA had a lower proportion of patients with a higher comorbidity burden (15.1% of TAA cases with ECI ≥3 vs 18.5% of AA cases with ECI ≥3, *P* < .001) (Table 1).

### Socioeconomic Factors and Hospital Factors

Socioeconomic factors are shown in the top half of Table 2. On univariate analysis of payor type, privately insured patients comprised a higher percentage of the TAA group (67.4%) than that of the AA group (49.1%, *P* < .001). With regard to median household income, the highest income quartile comprised the 30.5% of the TAA group compared with 18.4% of the AA group (*P* < .001). Conversely, only 18.0% of the TAA group was in the lowest income quartile compared with 24.2% of the AA group.

**Table 2.** Hospital and Socioeconomic Status of Patients Undergoing Total Ankle Arthroplasty (TAA) vs Arthrodesis (AA) for Ankle Osteoarthritis.

Variables	Total, n (%) (N = 9072; 100%)	Arthroplasty (TAA), n (%) (n = 2233; 24.6%)	Arthrodesis (AA), n (%) (n = 6839; 75.4%)	*P* Value
Payor information				<.001
Medicare	2922 (32.3)	467 (21.0)	2455 (36.0)	
Medicaid	1012 (11.2)	123 (5.5)	889 (13.0)	
Private insurance	4444 (49.1)	1497 (67.4)	4444 (49.1)	
Other	669 (7.4)	135 (6.1)	534 (7.8)	
Median household income				<.001
$1-$42 999	2019 (22.7)	396 (18.0)	1623 (24.2)	
$43 000-$53 999	2601 (29.2)	543 (24.7)	2058 (30.7)	
$54 000-$70 999	2380 (26.7)	586 (26.7)	1794 (26.7)	
$71 000 or more	1907 (21.4)	670 (30.5)	1237 (18.4)	
Hospital ownership				<.001
Government, nonfederal	1074 (11.8)	172 (7.7)	902 (13.2)	
Private, not-for-profit	7134 (78.6)	1819 (81.4)	5315 (77.7)	
Private, investor-owned	865 (9.5)	243 (10.9)	622 (9.1)	
Hospital bed size				<.001
00-99	1362 (15.0)	317 (14.2)	1045 (15.3)	
100-299	3037 (33.5)	869 (38.9)	2168 (31.7)	
≥300	4674 (51.5)	1047 (46.9)	3627 (53.0)	
Hospital type				<.001
Rural	764 (8.4)	130 (5.8)	634 (9.3)	
Urban nonteaching	2210 (24.4)	560 (25.1)	1650 (24.1)	
Urban teaching	6098 (67.2)	1543 (69.1)	4555 (66.6)	
Hospital region				<.001
Northeast	1493 (16.5)	318 (14.2)	1175 (17.2)	
Midwest	2491 (27.5)	625 (28.0)	1866 (27.3)	
South	3232 (35.6)	760 (34.0)	2472 (36.2)	
West	1855 (20.4)	530 (23.7)	1325 (19.4)	

Hospital factors are shown in the bottom half of Table 2. On univariate analysis of hospital bedsize, both TAA and AA were predominantly done in the largest hospital bed size category (53.0% and 46.9%, respectively). Conversely, the lowest percentage of TAA cases were performed at the smallest hospitals by bed size (18.0%), compared with 24.2% of AA cases. With regard to hospital type, TAA was performed at slightly higher rates in urban teaching hospitals (69.1%) than AA was (66.6%, *P* < .001). Across all hospital regions, TAA was performed less frequently in the Northeast than AA was (14.2% vs 17.2%), but was performed more frequently in the West than AA was (23.7% vs 19.4%, *P* < .001). Lastly, total charges of care were compared between the 2 groups. TAA patients demonstrated higher charges associated with their care compared with AA patients ($76 578.91 ± $47 062.60 vs $46 407.23 ± $29 935.53, *P* < .001).

### Multivariate Regression

A binary logistic regression was then used to assess patient, socioeconomic, and hospital factors independently associated with TAA utilization compared to AA. TAA utilization was found to be inversely associated with cumulative ECI (odds ratio [OR] = 0.92; *P* < .001).

**Figure 2.**
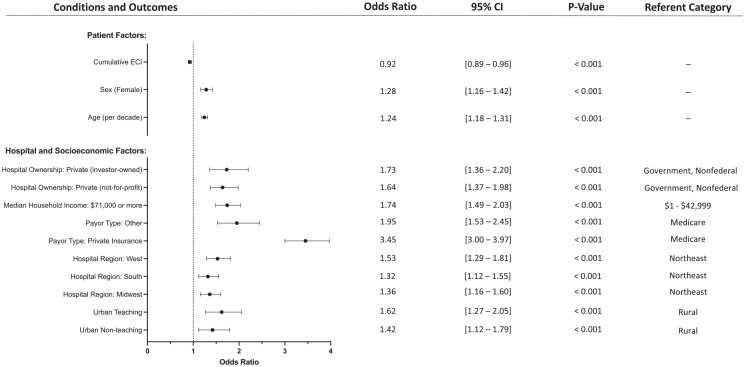
Forest plot depicting the significant variables from the multivariable regression on patient, hospital, and socioeconomic factors associated with utilization of ankle arthroplasty vs arthrodesis.

Conversely, TAA utilization was directly associated with the following covariates: age (per decade, OR = 1.24; *P* < .001), female sex (OR = 1.28; *P* < .001), urban nonteaching hospitals (relative to rural hospitals, OR = 1.42; *P* = .004), urban teaching hospitals (OR = 1.62; *P* < .001), the Midwest (relative to the Northeast, OR = 1.36; *P* < .001), the South (OR = 1.32; *P* < .001), the West (1.53; *P* < .001), private insurance (relative to Medicare, OR = 3.45; *P* < .001), other insurance (OR = 1.95; *P* < .001), increased median household income (relative to $1-42 999, $71 000 or more, OR = 1.73; *P* < .001), private not-for-profit hospital ownership (relative to government, nonfederal, OR = 1.64; *P* < .001), and private investor-owned (OR = 1.73; *P* < .001). These results with their accompanying CIs are shown in Table 3, and statistically significant findings are highlighted in Figure 2. The *P* value of the Hosmer-Lemeshow test was .945, indicating a good fit. Variance inflation factor values among individual covariates included in the regression (Table 2) ranged from 1.033 to 1.674, indicating relatively little collinearity.

**Table 3.** Patient, Hospital, and Socioeconomic Predictors of TAA Utilization, Identified Using Binary Logistic Regression Analyses.

Variables	OR (95% CI)	*P* Value
Age (per decade)	1.24 (1.18-1.31)	<.001
Sex (female)	1.28 (1.16-1.42)	<.001
Cumulative ECI	0.92 (0.89-0.96)	<.001
Hospital bed size		
00-99	Ref.	Ref.
100-299	1.17 (0.99-1.39)	.070
≥300	0.81 (0.67-0.97)	.021
Hospital type		
Rural	Ref.	Ref.
Urban nonteaching	1.42 (1.12-1.79)	.004
Urban teaching	1.62 (1.27-2.05)	<.001
Hospital region		
Northeast	Ref.	Ref.
Midwest	1.36 (1.16-1.60)	<.001
South	1.32 (1.12-1.55)	<.001
West	1.53 (1.29-1.81)	<.001
Payor information		
Medicare	Ref.	Ref.
Medicaid	1.23 (0.96-1.55)	.105
Private insurance	3.45 (3.00-3.97)	<.001
Other	1.95 (1.53-2.45)	<.001
Median household income		
$1-$42 999	Ref.	Ref.
$43 000-$53 999	1.00 (0.86-1.16)	.970
$54 000-$70 999	1.14 (0.98-1.33)	.097
$71 000 or more	1.74 (1.49-2.03)	<.001
Hospital ownership		
Government, nonfederal	Ref.	Ref.
Private, not-for-profit	1.64 (1.37-1.98)	<.001
Private, investor-owned	1.73 (1.36-2.20)	<.001

Abbreviation: ECI, Elixhauser comorbidity index; OR, odds ratio; TAA, total ankle arthroplasy.

## Discussion

End-stage ankle osteoarthritis is a common, debilitating condition that can be treated definitively with AA or TAA.^21^ Using a large, US national database, the present study determined that during the study period of 2016 to 2017, only 24.6% of eligible patients identified underwent TAA compared to AA. Multivariable analysis showed that several patient, socioeconomic, and hospital factors were independently associated with which treatment modality was employed. Although AA has traditionally been viewed as the gold standard treatment for end-stage ankle arthritis, recent advances in technology and surgical techniques have made TAA a more popular treatment option.^3,11,21^ Prior studies have shown that TAA may carry higher postoperative complications than AA; however, they also reveal that patients who undergo TAA tend to have superior clinical outcomes, reduced pain, more symmetric gait, and more natural postoperative arc of motion.^3,18,22,26^ Given the numerous purported benefits of TAA when compared to AA, it is notable that less than one-fourth of eligible patients with end-stage ankle osteoarthritis underwent the procedure.

Older patients were more likely to undergo TAA for end-stage ankle osteoarthritis than AA. Young, active patients are generally recommended to undergo AA because of minimal associated limitations or activity restrictions.^21,24^ Furthermore, past studies have revealed a positive effect of age on pain relief following TAA.^10^ In addition to age, female sex was associated with increased likelihood of undergoing TAA. Past studies have shown similar outcomes for men and women following TAA; however, women have been shown to have worse preoperative pain and functional assessments.^5,9^ As such, it is possible that women are favored for TAA because of greater perceived need. In contrast, patients with greater comorbidity burden were less likely to receive TAA over AA. Prior studies have shown significantly higher risk of implant failure in TAA patients with comorbid conditions such as rheumatoid arthritis.^17^ Past studies have also shown that patients who were obese or diabetic were more likely to undergo AA than TAA.^24^ Patient comorbidities can also have a negative impact on tissue healing following surgery, which is an important consideration in arthroplasty.^35^ Furthermore, comorbidities can pose an obstacle to patients’ completion of postoperative rehabilitation protocols, which are essential to proper recovery.

Regarding patient economic factors, patients living in a zip code with a median household income of $71 000 or more were significantly more likely to undergo TAA for end-stage ankle osteoarthritis. Previous studies have revealed patient income to be an important mediator of health care disparities.^7^ Past work in hand surgery has found that variations in the geographic density of specialist hand surgeons is closely associated with patient median per capita income.^25^ TAA is a highly technical operation, with surgeon experience being shown to strongly influence complication rates.^2^ Given this, specialists—and therefore specialized treatment options available to patients such as TAA—may be associated with patient income. In terms of patient insurance status, those with private insurance were significantly more likely to undergo TAA. Hospital charges for TAA are significantly higher than for AA, potentially serving as an incentive to perform TAA in patients with private insurance whose plans may reimburse more generously than Medicaid. Numerous studies have found barriers to care among patients with Medicaid insurance, including increased travel time to care and worse preoperative health.^14^

Urban teaching and urban nonteaching hospitals were significantly more likely to use TAA than were rural hospitals. One 2016 study analyzing TAA utilization from 2003 to 2011 using a large national patient sample found that patients in rural areas were less likely to receive TAA in 2003; however, this disparity had resolved by 2011.^31^ In contrast, a 2014 study found that private urban and academic hospitals were significantly more likely to perform TAA as opposed to AA when compared to rural hospitals.^24^ Given that TAA is a highly technical surgery, it is possible that orthopaedic surgeons who perform this procedure concentrate in large, high-volume urban centers with greater access to the training, staff, and other resources necessary to aid in its success. Furthermore, patients may actively seek out large urban hospitals with greater perceived reputation and brand recognition for what they perceive to be more complex procedures, such as TAA.^33,38^ In addition to urban teaching and nonteaching hospitals, private, not-for-profit, and private, investor-owned hospitals were more likely to offer TAA. It is possible that private hospitals are better equipped with more specialized orthopaedic surgeons as well as the resources required to carry out complicated procedures such as TAA.

Lastly, there was significant variation in the utilization of TAA by geographic region. Several factors may influence regional variation in the employment of TAA over AA for treatment of end-stage ankle osteoarthritis, such as varying geographic density in fellowship-trained orthopaedic surgeons, differential access to equipped surgical centers, and varying accessibility of postoperative recovery resources. Regional training networks may also induce variation in geographic practice patterns. Similarly to the present study, previous works in the orthopaedics literature have identified regional variations in treatment regimens and procedure utilization.^27,36^

There are several limitations that should be noted. Perhaps the most important limitation is that the NASS database only captures ambulatory surgery encounters at hospital-owned facilities. As such, instances of TAA and AA performed at privately owned or freestanding ambulatory surgical centers, or those performed inpatient, are excluded from this analysis. No studies explicitly characterize the proportion of TAA and AA performed inpatient vs outpatient; however, general trends suggest that outpatient approaches to both procedures are growing increasingly popular.^23^ Although a large proportion of these surgeries are performed in the inpatient setting, the increasing safety and efficacy of outpatient TAA has been demonstrated and will likely increase in volume over time, giving this research increasing relevance.^16^ Additionally, the NASS database fails to account for several personal factors that may influence a patient or surgeon to opt for TAA or AA. For example, the database cannot provide insight into patient treatment preferences, perceived ability to comply with postoperative rehabilitation requirements, or understanding of treatment options. Further, NASS does not allow for analysis of patient race/ethnicity, which could influence the utilization of TAA vs. AA due to systemic factors or bias. With regard to health care cost, the NASS database provides total charge figures rather than total cost, which are known to vary and be an inexact marker of economic efficiency.^8^ From a provider perspective, the NASS database cannot identify surgeon preferences in comfort performing TAA or AA. Lastly, the NASS database only allows generation of ECI based on visit-level claims rather than claims history, potentially underestimating the overall comorbidity burden of patients in the present study.

Ultimately, using a large nationally representative cohort, the present study revealed that 24.6% of operatively managed end-stage ankle osteoarthritis cases were treated with TAA during the time period studied. Given that the present study identified several patient, hospital, and socioeconomic factors independently associated with TAA utilization, it is highly likely that other factors may influence the choice of one treatment over the other for this patient population. Surgeons should be aware of potential health care disparities when choosing treatment options to enable fair and optimal treatment for all patients. These results may guide future clinical and policy recommendations for ensuring equitable access to TAA despite possible socioeconomic barriers to care.

## Supplemental Material

sj-pdf-1-fao-10.1177_24730114231218011 – Supplemental material for Differential Utilization Patterns of Total Ankle Arthroplasty vs Arthrodesis: A United States National Ambulatory Database Analysis Click here for additional data file. Supplemental material, sj-pdf-1-fao-10.1177_24730114231218011 for Differential Utilization Patterns of Total Ankle Arthroplasty vs Arthrodesis: A United States National Ambulatory Database Analysis by Michael R. Mercier, Philip P. Ratnasamy, Nicholas S. Yee, Brandon Hall, Christopher Del Baso, Mohammed Athar, Timothy R. Daniels and Mansur M. Halai in Foot & Ankle Orthopaedics
